# Strategies Used by Musicians to Identify Notes’ Pitch: Cognitive Bricks and Mental Representations

**DOI:** 10.3389/fpsyg.2020.01480

**Published:** 2020-07-07

**Authors:** Alain Letailleur, Erica Bisesi, Pierre Legrain

**Affiliations:** ^1^CNRS UMR 8131, Centre Georg Simmel Recherches Franco-Allemandes en Sciences Sociales, École des Hautes Études en Sciences Sociales (EHESS), Paris, France; ^2^CNRS UMR 3571, Paris, France; ^3^Unité Perception et Mémoire, Institut Pasteur, Paris, France

**Keywords:** mental anchorpoint, mental representation, multimodal music perception, introspection, phenomenology, note pitch

## Abstract

To this day, the study of the substratum of thought and its implied mechanisms is rarely directly addressed. Nowadays, systemic approaches based on introspective methodologies are no longer fashionable and are often overlooked or ignored. Most frequently, reductionist approaches are followed for deciphering the neuronal circuits functionally associated with cognitive processes. However, we argue that systemic studies of individual thought may still contribute to a useful and complementary description of the multimodal nature of perception, because they can take into account individual diversity while still identifying the common features of perceptual processes. We propose to address this question by looking at one possible task for recognition of a “signifying sound”, as an example of conceptual grasping of a perceptual response. By adopting a mixed approach combining qualitative analyses of interviews based on introspection with quantitative statistical analyses carried out on the resulting categorization, this study describes a variety of mental strategies used by musicians to identify notes’ pitch. Sixty-seven musicians (music students and professionals) were interviewed, revealing that musicians utilize intermediate steps during note identification by selecting or activating cognitive bricks that help construct and reach the correct decision. We named these elements “mental anchorpoints” (MA). Although the anchorpoints are not universal, and differ between individuals, they can be grouped into categories related to three main sensory modalities – auditory, visual and kinesthetic. Such categorization enabled us to characterize the mental representations (MR) that allow musicians to name notes in relationship to eleven basic typologies of anchorpoints. We propose a conceptual framework which summarizes the process of note identification in five steps, starting from sensory detection and ending with the verbalization of the note pitch, passing through the pivotal role of MAs and MRs. We found that musicians use multiple strategies and select individual combinations of MAs belonging to these three different sensory modalities, both in isolation and in combination.

## Introduction

Scientific studies on human cognition reveal that the brain, including its sensory inputs involved in cognitive functioning, has a highly complex structural and functional architecture. The objective of deciphering such a functional architecture is therefore extremely ambitious. The modern period of this search was in fact initiated in the second part of 19th century, with the founders of the experimental psychology, W. Wundt in Germany and W. James in the United States (Thomas, 2019). Since, considerable scientific progress have been made, mainly in the field of experimental neurosciences (for a review, see Dehaene and Changeux, 2011). The causal link between complex cognitive processes and the regulation of gene expression, including epigenetic mechanisms, is now being investigated, especially about learning and memory processes or child behaviors (for reviews, see Borrelli et al., 2008; Grigorenko et al., 2016; O’Donnell and Meaney, 2020). In addition, a very fertile interdisciplinary field of research has emerged over the last years, favoring the combination of approaches and concepts from the humanities, cognitive sciences and experimental neurosciences. There is undoubtedly a desire to make the acquired knowledge coherent that is, for example, linking the functional description of the brain, jointly from a mental and a neural point of view. However, this task proves difficult, as it attempts to join methods often considered as opposed. Such a problem emerges in particular when one juxtaposes human sciences to natural sciences (Varela et al., 2001; for contribution of philosophy to cognitive sciences, see also Laplane et al., 2019). In a *bottom–up* experimental approach, neurosciences tackle the functional description of the brain by proposing methods aimed at establishing the neural correlates of mental states with the results of behavioral experiments and neural mapping. But in the majority of cases, these studies are still far from being able to concretely grasp the essence of *the matter of thought*. We note that this follows from methodological difficulties in developing reliable tools likely to validate such direct experience.

Hence, with the aim of tackling directly the study of the matter of thought, we opted for a holistic method. We followed a phenomenological approach based on introspection, i.e., we started from the mental point of view rather than from the neuronal substrate material, following an exploratory interview method. After a first series of interviews, we gradually refined and stabilized our protocol. We narrowed our observations as much as possible to a very specific task in the field of auditory cognition, ending up with the identification of the pitch of one or a few musical notes by students or professional musicians.

Since the beginning of the 20th century, Husserl (1928) adopted a phenomenological perspective to face reality from a subjective point of view. Phenomenology is a milestone in cognition because it regards “subjective experience as a continuous process that is lived from within” (Ollagnier-Beldame, 2019). In doing so, through introspection, it allows identification of those discrete mental objects that are likely to be of interest for the research purposes: “Experience is the familiar knowledge we have of our mind and our action, namely, the lived and first-hand testimony we have about it” (Depraz et al., 2003). One might advance that phenomenological examination of human thoughts can not only bring to light mental contents of which we are not aware, but also reveals how to search for them: “Our lived experience being what is closest to us, the most intimate, we do not imagine that any particular work is necessary to become aware of it” (Petitmengin et al., 2015; see also Vermersch, 2004; Petitmengin and Lachaux, 2013). Although phenomenology can concretely bring original and innovative inputs on this side, this method, which corresponds to a *top–down* experimental approach, presents in return a major drawback: it is solely based on subjective therefore potentially misleading pieces of evidence (Johansson et al., 2005). The present study aims at limiting such methodological difficulties by collecting interviews based on introspection on a large number of subjects, that is: by comparing and cross-referencing the descriptions provided by a wide heterogeneity of participants, we aim at identifying at the same time substantial elements of individuality and emerging generality.

Music perception mobilized researchers since the end of the 19th century. For example, Stumpf proposed the concept of “*Verschmelzung*” (in English “amalgamation”) of sounds: as we hear a mixture of complex sounds, we may, on the one hand, switch between apperception of many, even all, of their constituents; on the other hand, complex sounds may be perceived as a highly integrated whole (Stumpf, 1890). Nowadays, a broad scientific community is studying the neural bases of musical listening (Allen et al., 2017; Graves and Oxenham, 2019; for reviews, see Zatorre and Krumhansl, 2002; Levitin, 2012; Peretz et al., 2015). In this study, we will focus on the mental strategies adopted by musicians in the process of note pitch recognition. This represents the starting point for our ongoing project, whose aim is to relate these mental elements to the corresponding neural networks activated in the brain that are being currently studied directly from a neural perspective (Brauchli et al., 2019; Burkhard et al., 2019; Leipold et al., 2019; McKetton et al., 2019).

The scientific literature focusing on absolute pitch reveals the existence of a very wide range of hearing abilities (Takeuchi and Hulse, 1993; Levitin and Rogers, 2005; Schneider et al., 2005; Hsieh and Saberi, 2008; Deutsch, 2013; Kim and Knösche, 2017). We chose not to limit ourselves to individuals with self-certified absolute pitch, preferring to gather more information on this ability from the study itself: for example, in case of failure or difficulties in the task, a reference note was proposed to the participant. This methodological approach has the advantage of allowing identification of a wide range of hearing abilities, from the perfect ability of those with absolute pitch to recognize any non-contextualized note, to the contextual ability to identify an auditory stimulus with the assistance of an anchoring or reference sound.

The introspective method to gain insights into the contents of thoughts has been used by researchers exploring the theory of mental management (La Garanderie, 1989), elicitation interviewing (Vermersch, 1994), or, more recently experiential phenomenological interviewing (Vion-Dury and Mougin, 2018) and micro-phenomenology (Petitmengin et al., 2019). The technique of elicitation interviewing was the first to address a genuine search for objectivity, focusing on the “scientification” of interviews by directing questioning toward the implicit elements of the experience of action (Vermersch, 1994). More specifically, micro-phenomenological analysis consists in the identification of structural statements as minimal units of meaning, potentially referring to or instantiating descriptive categories (Petitmengin et al., 2019).

In order to facilitate description of the mental texture at the moment of note identification and aiming at the detection of specific and individual cognitive bricks, we questioned musicians to introspectively relate the strategies with which they mentally proceeded, by asking them to describe, as precisely as possible, the mental processes they were conscious of going through. We then processed the data in two stages: qualitatively, by collecting information from each musician in order to establish an inventory of the detected strategies; quantitatively, by cross-referencing these results by means of a statistical approach. This made it possible to categorize the different mental strategies and to propose an interpretation based on specific phenotypic signatures.

## Materials and Methods

### Participants

Over eighty subjects were initially contacted. Among them 67 people were ultimately included: 28 professional musicians (4 females and 24 males, between 27 to 84 years old), and 39 Conservatory students (18 females and 21 males, between 6 to 31 years old) (Table 1). Professional musicians were recruited through professional relationships in Conservatories, orchestras or choirs. Students were recruited from three different Conservatories in the Paris region at various degrees of training, covering first and second French Conservatory cycles (corresponding to 4-years training for students aged 6–10 years and 10–15 years, respectively), except for P122 and P144 who attended a third cycle (aged usually above 16 years). Participants were initially contacted in person, by phone or by email, and informed about the aim of the research that is how to improve musical hearing through a better understanding of the underlying cognitive processes. All participants provided their consent to participate in the study before the beginning. In the case of minors, parents provided consent on their behalf. All data and reports were anonymized in accordance with the Good Practice Guidelines provided by the research institution EHESS, which also formally agreed on the proposed protocol. No formal agreement by an ethical committee was requested at the time of the interviews.

**TABLE 1:** Participant list.

Participant	Gender	Expertise	Age	Musical Practice	Comments
P101	M	PRO	65	Choir conductor	Pilot group
P102	M	STU	11	Piano	
P103	F	STU	12	Guitar	
P104	M	STU	11	Piano	
P105	F	STU	16	Violin	
P106	M	STU	12	Guitar	Out
P107	F	PRO	59	Piano/Composer	
P108	M	STU	16	Violin	
P109	F	STU	14	Piano	
P110	F	PRO	45	Singer	Pilot group
P111	M	STU	11	Piano	
P112	F	PRO	29	Composer	Pilot group
P113	F	STU	13	Flute	
P114	M	PRO	84	Piano/Composer	
P115	F	STU	9	Guitar	Out
P116	F	STU	13	Piano	
P117	M	STU	8	Undetermined	
P118	F	STU	8	Piano	
P119	M	STU	15	Piano	
P120	M	PRO	50	Percussion	
P121	M	PRO	59	Conductor	
P122	M	STU	12	Cello/Piano	
P123	M	PRO	37	Piano/Composer	Pilot group
P124	M	PRO	42	Piano	
P125	M	PRO	45	Trumpet	Out
P126	M	PRO	30	Piano	Pilot group
P127	M	PRO	73	Composer	Out
P128	M	PRO	37	Piano/Viola	
P129	M	STU	7	Clarinet	
P130	M	PRO	42	Cello/Conductor	
P131	M	PRO	43	Choir conductor	
P132	M	STU	24	Clarinet	Pilot group
P133	M	PRO	36	Piano/Composer	
P134	M	PRO	63	Composer	Out
P135	F	STU	12	Piano	
P136	M	PRO	79	Piano/Composer	Pilot group
P137	M	STU	6	Undetermined	Out
P138	M	PRO	65	Choir conductor	
P139	M	PRO	56	Cello	
P140	F	STU	14	Guitar	
P141	F	STU	13	Guitar	Out
P142	M	STU	20	Saxophone	
P143	F	STU	10	Flute	
P144	F	STU	17	Cello/Horn	Pilot group
P145	F	STU	9	Viola	
P146	M	PRO	32	Viola	Out
P147	M	PRO	36	Balafon	Out
P148	M	PRO	31	Piano/Composer	Pilot group
P149	M	STU	6	Piano	
P150	M	STU	14	Violin	
P151	M	STU	11	Guitar	
P152	M	PRO	39	Flute	Out
P153	F	STU	9	Violin	
P154	M	STU	14	Piano	
P155	M	STU	31	Guitar/Trumpet	
P156	F	STU	16	Saxophone	
P157	F	STU	7	Cello	
P158	F	STU	13	Piano	
P159	M	STU	15	Bass guitar	Out
P160	F	STU	15	Piano	
P161	M	PRO	53	Piano/Composer	
P162	M	STU	10	Cello	
P163	F	PRO	27	Organ	Pilot group
P164	M	PRO	40	Organ/Composer	
P165	M	PRO	52	Violin	
P166	M	STU	22	Violin	
P167	M	STU	6	Percussion	

*Participants’ profiles: gender (M or F), musical expertise (STU: student at the Conservatory, PRO: professional musician), age at the time of the interview, musical practice (undetermined: non-constant practice). In the sixth column, we indicated the role of the participant in the study: “pilot group,” the participant was selected for the initial investigation on a limited group of 10 musicians; “out,” the interview was not suitable for quantitative analysis and was therefore discarded.*

### Materials: Stimuli

**FIGURE 1:**
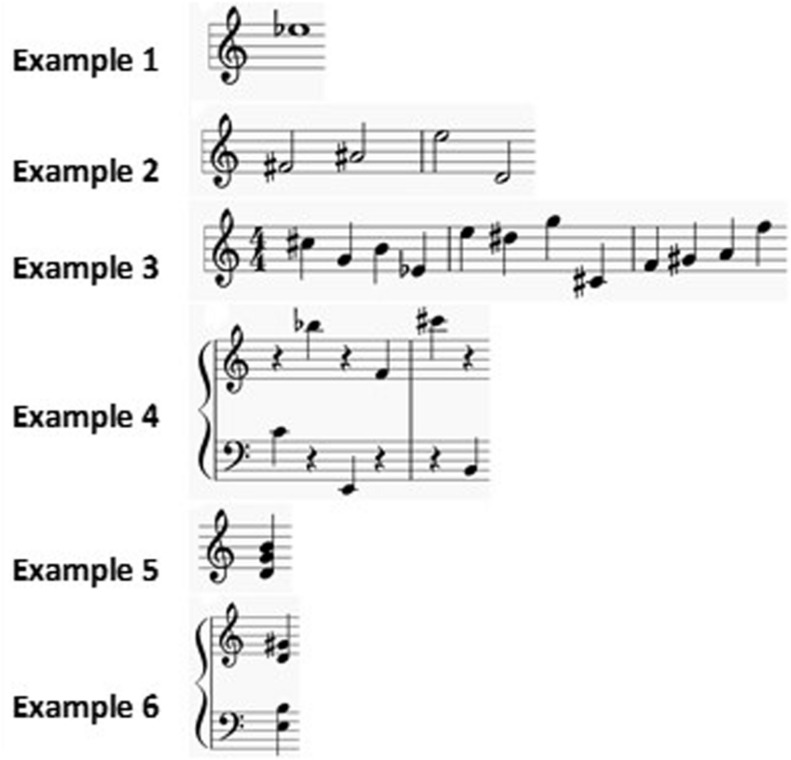
Examples of auditory stimuli. Examples of auditory stimuli corresponding to different levels of complexity. Example 1: E flat played without a reference note. When the participant asked, it was played again preceded by a reference A4. Example 2: four notes that might be perceived in a tonal or non-tonal context. Example 3: twelve notes in a non-tonal context. Example 4: six notes distributed over a large ambitus, in a non-tonal context. Example 5: a major chord in its second inversion. Example 6: a dominant seventh chord in its fundamental state.

One or a small number of notes of various pitches were provided to each subject. In the case of several notes, they were presented in sequence (melodies) or in superposition (chords). Representative examples are presented in Figure 1. The notes were played on the piano (various types), or on the computer with a piano timbre; in the latter case, the piano sound was synthesized by means of the Finale music editing software (Version, 2012a, MakeMusic, Boulder, United States^1^). One participant (P165), who played the violin, complained that identification of notes at the piano was too difficult; as requested, the notes were then played at the violin. When required, a reference note (generally, an A4 which is the common reference used in French Conservatories) was provided.

### Procedures: Interviews and Questionnaires

All interviews were performed by AL, a teacher and professional musician, as a contribution to his Ph.D. research. Interviews took place at Conservatories. They lasted between 30 min and two and a half hours. For two minor students only, a parent was present during the interview. On a whole, 80 interviews were performed, most of them during a 3-year period (2005–2007). Fifteen interviews were performed before 2000 and served to refine the procedure (see below); seven interviews were performed later (2015–2016). One participant declined to participate in the study and another dropped out after completion of the interview; both argued that the questionnaire was intrusive and potentially damaging for their image. All documents relative to these interviews have been deleted. At the beginning of the interviews, participants were asked to answer simple questions about their musical background (expertise, instrumental practice, etc.). All the interviews were recorded in audio format and faithfully transcribed into text files. Participants were not asked to read, comment or correct the texts. In some cases, a few comments were added during interviews and identified as such in the written transcription. For four musicians (P112, P114, P123, P164), several sessions took place. In one case (P107), the interview was summarized a posteriori.

Interviews were conducted following an approach based on introspection. The study started with a quite long exploratory stage during which the method was progressively stabilized, being also influenced by interview methods proposed in the nineties (La Garanderie, 1989; Vermersch, 1994). However, we didn’t strictly follow one or the other of these methodologies, but rather explored an original approach facilitating the communication between the subjects and the experimenter about the involved mental processes. Examples of such interviews are reported in AL’s Ph.D. thesis (Letailleur, 2017). Our interviewing method presents some similarities with the introspective interviewing techniques described in the literature, such as the renewed version of elicitation interviews (Depraz et al., 2003; Vermersch, 2009), experiential phenomenological interviews (Vion-Dury and Mougin, 2018), or microphenomenological interviews (Petitmengin et al., 2019).

The main task of each interview was to identify individual notes. The interviews systematically started with identification of individual notes, isolated from any context (Figure 1, Example 1). Whenever possible, i.e., when the musicians easily managed to identify isolated notes, the interview moved forward including more complex stimuli (Figure 1, examples 2–6). Our aim was to propose a task systematically compatible with participants’ assessed expertise: when deemed appropriate, this led us to identification tasks of higher complexity. Participants were always asked to describe the mental paths they followed during note identification. Our objective was not to test the expertise of the participants, but to collect information and description on the mental elements activated during the identification process for which their expertise contributed. The whole procedure was calibrated to ensure, as far as possible, that the complexity of the task was compatible with participants’ expertise. For this reason, a reference note was offered upon request from participants who did not have absolute pitch. In the vast majority of cases, musicians were able to identify the notes and describe the mental strategies adopted in the process of identification. For those cases (mainly young students) where correct identification was not achieved, participants were not warned of their mistakes and the interview nevertheless continued with unchanged questions.

### Analytical Procedures

First, a preliminary, qualitative, overview of the entire dataset of interviews allowed us to extract pieces of evidence for various mental strategies during the process of note recognition. Different mental elements of small complexity were identified. We named such elements “mental anchorpoints” (MA). MAs are associated, one way or the other, with the mental representations (MR) of the note’s names, i.e., the inner elements which come to the mind to explain the external signal and ascribe it a meaning. Second, we carried on systematic quantitative analyses of interviews, by extracting and classifying the text elements corresponding to MAs. We started with a pilot study on ten interviews, where annotations for MA and MR were independently carried on by two of us – AL and PL (who are both French native speakers), by means of the QDA Miner 5 software (Provalis Research, Montreal, Canada)^2^ – a tool equipped by a very efficient interface for coding, extraction, and qualitative and quantitative analysis of written text. The internal consistency of our independent annotations and consequent reliability of the method was assessed by means of the Cohen’s kappa test (McHugh, 2012; see section “Pilot Study on a Set of 10 Musicians” for results). Further on, AL and PL jointly annotated the occurrences of MAs for the complementary group of forty-six participants. Consistency between the two annotation methods was finally assessed by means of the Kolmogorov–Smirnov test (Howell, 2013; see section “Complete Analysis on the Whole Group of 67 Musicians: Selection of MAs and Effect of Expertise” for results).

## Results

### Qualitative Analysis

**FIGURE 2:**
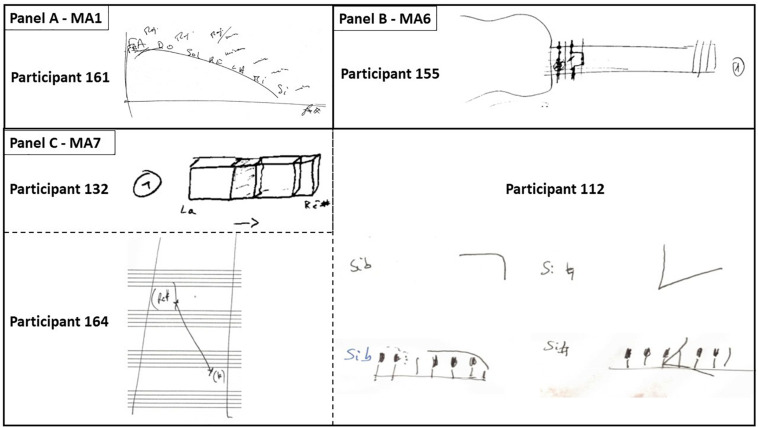
Drawings made by some participants to illustrate their mental anchorpoints. **(A)** Participant 161 made a drawing to explain how he heard the names of notes (MA1) at different loudness, from very loud to very soft (quoted “*Max*” and “*minim*” in the drawing) along an ascending pattern of fifths (*Fa Do Sol Ré La Mi Si*; F C G D A E B). **(B)** Participant 155 drew a guitar with the position of the fingers playing specific notes (MA6). **(C)** Participants P112, P132, and P164 drew specific diagrams (MA7) to explain their mental representations of notes.

Most of the interviews revealed that musicians were not aware of the strategy or method they used to identify the pitch of a note. Once the note name was provided (e.g. “*It’s an E flat*”), the question “*How do you know this?*” was systematically asked. A few musicians were spontaneously able to describe their strategies, for example: “*Because I “heard*” – or “*saw*,” etc. – *something in my head.*” Most musicians found themselves unable to provide such an explanation and just answered “*It’s obvious*”. In such cases, the interviewer insisted by inducing introspection on the process of note recognition: “*Can you try to describe what happened in your mind during identification*?”. In the end, the mental processes leading to the identification of notes were reported by participants through a wide variety of mental descriptors that were interpreted by the participants themselves as dynamic supports in the act of identifying the note. In some cases, participants illustrated their anchorpoints by means of drawings, as reported in Figure 2.

#### Examples of Descriptors as Mental Anchorpoints for Note’s Recognition

The result of gathering evidence from many musicians, revealing their cognitive paths to note identification, led us to consider each of these elements of thought playing the role of a cue, by contributing to the recognition of the perceived note(s). Such descriptors emerged as the smallest mental elements that could be detected by means of introspection. We formulated the concept of “mental anchorpoints” as the minimal structures detectable by a subject by introspection, in association with a perceptual recognition process. Such MAs were described by participants in several ways. For example, participant P125 indicated that the “sounding” nature of his MA consisted of a fusion between two timbres: “*It is about an inner voice, a timbre, a color, halfway between the human voice and a trumpet sound.*” Participant P138, concerning the identification of the note F sharp, insisted on that he could hear three syllables melted into a single one: “*As well as I hear only a single sound, I hear only one syllable. Sounds are inseparable. For me, it’s a global perception: I do not hear two or three syllables. I get a simultaneous sound impact, and even if I would require three syllables to express this, in my mind it’s as if there were only one: if I could pronounce the three syllables at the same time, I would pronounce them at the same time.”*

Mental anchorpoints, as a support for recognizing a low-complexity perceptual object, appeared then to work as key elements of an effective cognitive strategy: sometimes as immediate grasps, that might even be ignored so far by the musicians themselves, or as a micro-reasoning requiring several seconds. The idea that MAs play a strategic role is also reflected by the fact that it was possible to establish a strong correspondence between their use and different ways of ascribing a meaning to perceptual features. To provide an example, we observed a significant consistency between the specific adopted strategy and the type of musical hearing (relative or absolute pitch). Musicians with relative pitch failed to identify an isolated note, and asked to hear a reference note first. These musicians said that they were to mentally compare the gap between the two notes with an interval stored in their memory. This revealed a relationship between the employed strategy and relative pitch, since the procedure for identification indicated a sort of measurement of the distance between two references. Those having absolute pitch identified the note without any prior reference. They said for example that they mentally felt the gesture that should be performed with their instrument or phonatory system to produce that pitch. This strategy is compatible with direct pitch recognition without any comparison between two sounds. But this was not always the case. Here is a concrete example: participant P164 never requested a reference note to identify a note in isolation. However, in some situations, he mentally imagined a reference note A4 and compared it with the external sound he was physically perceiving. He then had a kind of “absolute” approach, typical of perfect pitch, consisting in not-need of any external reference but proceeding nevertheless by “relative” comparison of two elements: recognizing a first (internally devised) note before identifying the second (really heard) one. It should be noted that, as a first approximation, this approach did not seem to require more time than direct identification. Many musicians with absolute pitch showed different abilities to stock internal references in their memory over time. Participant P123 specified, for example, that he might stay for several days, even weeks, without listening to music, still maintaining his capability to identify the notes in an absolute way. Differently, participant P139 said that he needed to “reactivate” this skill one or more times a day.

All these descriptions suggested a direct relationship, connecting the MAs with the response(s) in the process of identification. Here are two additional examples:

(a) Participant P164 did a comparison between two sounds. When he described his MA, he evoked the distance between the sounds and said that it was the relative position of the second “luminous point” that allowed him to identify the note in question (Figure 2C): “*In fact, for this note, I did not see a keyboard, but an interval, a distance between two sounds (…). A kind of black background, with the interval between the notes on the keyboard like a [space] distance on a neutral background (…) I felt the mental distance of five keys.*”
(b) As for participant P163, she often heard internally pronounced syllables such as C (“Do” in French) or E (“Mi” in French), which put her on the track of naming the note.

#### The Mental Strategies

From participants’ reports, many situations emerged where the MAs interacted with each other, spreading out or merging together. Processes in which the identification occurred through combination of several MAs were regularly described. In this situation, each MA provided only one piece of information, and it was therefore from the combination of several complementary elements that pitch recognition was achieved. Thus, for example, participant P163 needed to focus on three aspects: (1) internally heard vowels, which provided information on the name of the note (“*The note said to me “mi”* [E in French]); (2) the specificity with which the ending vowel of the note name was pronounced (the ending *i* was pronounced with a rather dark color: “*It was a bit like a “mu*””), which put on the trail of detecting an alteration (i.e., an E flat and not an E natural), and finally (3) the blurred vision of a piano: “… *and I superimposed the overall view of the piano to detect which E it was about.*”

The obtained results suggested that this diversity of strategies was far from being concerned with only the distinction between the two main types of musical ear, i.e., absolute versus relative pitch. Indeed, participants mentioned explicitly the contribution of their intentional aim during note recognition especially for complex sounds (more than one note; see Figure 1, Examples 5 and 6). Here are two descriptions of contrasting mental procedures: (i) “*I search, note by note, for the different elements of a chord*,” or alternatively (ii) “*I identify a chord first as a whole.*” Some musicians might use either strategies, for example P123. These examples suggest that, depending on what attention was focused on, strategies could differ, still using the same MA. Another illustration of the role of intentional aims during note identification is: most musicians mentally identified enharmonic pitches by naming the notes regardless of the harmonic context (for example, the systematic choice of a G-sharp and not an A-flat), mentioning, later on if applicable, the odd naming they came up with.

Finally, it was often through the aggregation of several MAs that musicians were allowed to make up a mental representation, that is to say the complete identification of the perceived note. Although MAs might be related to different sensory modalities (see below), the so formed MRs were always related to a single primary sensory modality – according to what all the participants declared: “I” mentally “see”, “hear” or “feel”.

This first qualitative analysis provided evidence that musicians didn’t used the same strategies or MAs to identify pitch. However, it also suggested that – while a wide range of descriptions was provided – the underlying mental processes were not so numerous. Consequently, we conducted a second, more focused, qualitative analysis on all available interviews in order to relate participants’ MAs to a limited number of categories and to identify a minimal repertoire of anchorpoints.

#### A Repertoire of Mental Anchorpoints for Pitch Recognition

The 11 resulting categories of MAs are listed in Table 2, along with a short description. In the following paragraphs, we will provide a detailed description of each of them.

**TABLE 2:** Categories of mental anchorpoints.

Mental sensory modality	Label	Mental strategy
Auditory	MA1	“Hearing” the name or part of the name of a note
	MA2	Recognition of formally learned intervals or sound patterns that combine several notes
	MA3	Search for note pitches by the use of rising or descending scales
	MA4	Note recognition by association of pitch with specific auditory hues, or the notion of “timbre-pitch”
Visual	MA5	“View” of the note inside the stave, or “view” of the name of the note
	MA6	“View” of a musical instrument or “view” of the body position required to play a note
	MA7	“View” of individually contrived specific patterns that correspond to a note or to a group of notes
	MA8	“View” of a color or matter associated to the note pitch
Kinesthetic	MA9	Feeling of a vocal gesture associated to the production of a note
	MA10	Feeling of an instrumental gesture associated to the production of a note
	MA11	Other miscellaneous feelings specific to a note

##### MA1: “Hearing” the name or part of the name of a note

This MA depends on the language of the interview (in our case, French). Musicians had various uses of this anchorpoint: indeed, descriptions focused on different aspects such as the specific location of the sound or the timbre with which the words were *internally* pronounced (being an entire word or part of it). It was the focus on the last vowel, for example, that made it possible to define the role of this MA. Some musicians, like P148 and P162, mentally “heard” only the final vowel of the note’s name. Both admitted to be confused between the notes *mi* and *si* (E and B in English), as well between *fa* and *la* (F and A), since their names ended with the same sound – a quite surprising confusion for an expert musician. The same didn’t happen, for example, to participant P139, who was able to “hear” the names of the notes in their entirety. Intriguingly, for some participants the sound location (i.e., where, in the real space, the name of the note was “heard” to be pronounced, as a kind of “spatial mapping”) was a very prominent mental strategy. For example, P123 “heard” the names of notes; in case of doubt, for example in the presence of alterations, he could distinguish between different possibilities as, he said, the sound sources “*generating different names*” were “*located in different positions.*” P123 used sound location for MA1 very frequently. Besides him, we found trace of such usage also in other interviews, for example in the case of P126. For P161, some notes might “*pronounce their names*” stronger than others depending on the pitch from the “*very loud*” *Fa* (F) to the “*almost inaudible*” *Si* (B) along an ascending circle of fifths, as drawn by the participant himself (Figure 2A).

##### MA2: Recognition of formally learned intervals or sound patterns that combine several notes

Here, we are talking about the recognition of groups of notes in relation to each other, which form a recognizable sound pattern. This MA, widely used, was exploited in two different and possibly complementary ways. In the first case, it concerned recognition of learned and memorized intervals such as the third, the fifth, etc. In this case, anchorpoint MA2 was used to recognize notes played both in sequence (melodies) and in superposition (chords). It essentially corresponded to a strategy associated with relative pitch. For example, participant P136 said: “*The intervals, (…) I recognize them in any case, for all instruments.*” In the second case, MA2 corresponded to direct recognition of conventional chords such as the major triad, the minor triad, the dominant seventh, etc. This kind of instantaneous grasp is typical of harmonic pattern recognition (Bougeret, 2005), and might be motivated by its immediate effect of simplifying the task. As P114 indicated: “*It is as if we had a memory of thousands of chords, and those we hear were identified by means of those of which we had memory*.” This MA was used in association with both absolute and relative pitch, although in different ways. Its usage seemed to be a consequence of an intensive musical training.

##### MA3: Search for note pitches by the use of rising or descending scales

The identification of a note started from a well-known reference pitch: the musician mentally sang or heard sounds with the purpose of counting the steps needed to reach the pitch under identification. As an example, student P102 said: “*I rose from C to B; I climbed C, D, E, F, G, A, B.”*

##### MA4: Note recognition by association of pitch with specific auditory hues, or the notion of “timbre-pitch”

This MA can be intended in analogy with a visual color (although it refers to a non-visual aspect, being a sort of auditory hue), associated to each note or chord. For most musicians, this anchorpoint seemed to be related to the wavelength of the fundamental, perceived as a unique signature; for some of them (we detected five participants), its usage was closely associated to the timbre of the sound, i.e., sounds containing a minimal number of higher harmonic frequencies and produced by well specific instruments (e.g., a violin for P165). Participant P131 stated: “*For me, the note itself has a particular timbre, a particular color, an E does not sound like a D, and if you play a note on the piano, it’s above all the timbre of that note that will provide me its name”*. For some musicians, pitch identification from the human voice (P163) or from a computer sound (P131) was impossible. Such individual diversity suggested a cognitive signal processing of the sound, whose complexity went far beyond the simple recognition of the fundamental pitch. This reminds to experimental studies comparing humans and macaques on the different functional organization of the auditory cortex for the treatment of the musical stimuli (Norman-Haignere et al., 2019). Some musicians used this anchorpoint also in the simultaneous recognition of several notes, without necessarily resorting to a previously learned music signature (like classified chords, perception of the difference between a perfect fourth and a major third, etc.).

Note that although the boundaries between MA2 and MA4, as well as between MA2 and MA3, are quite clearly defined, musicians demonstrated that they were able to use these anchorpoints also in combination, which makes their distinction less prominent. Note also that the distinction between MA2 and MA4 is particularly delicate, since the “color” of a chord – a feature expressing its timbral signature depending on the balance between the harmonics – can be either memorized as such or associated with previously learned music concepts.

##### MA5: “View” of the note inside the stave, or “view” of the name of the note

In this case, the notes were placed inside the stave, or their names “appeared” in full letters. For example, the young student P153 said: *“Yes, I saw the written word…, since when I think to the note, I don’t know why, in a solfeggio lesson, sometimes, I [mentally] write it… sometimes I think about it…”*. In the first case, the key could also appear, and the ambitus was also somehow taken into account (bass key for low pitches, violin key for high pitches). In this regard, P105 said: “*I imagine how the notes could be positioned (…) I see a stave with a key of Sol, an A, and then I see the notes going down…”*

##### MA6: “View” of a musical instrument or “view” of the body position required to play a note

Depending on the instrumental practice of the musician, a musical instrument appeared – a keyboard, a guitar fretboard, the clarinet keys, etc. – with the position of the fingers properly associated with the note. In some cases, participants provided a drawing of such mental images (e.g., P155, Figure 2B).

##### MA7: “View” of individually contrived specific patterns that correspond to a note or to a group of notes

This MA was used to identify a note without any obvious reference (for instance, absolute pitch), to identify a note in association with a reference note (either played or not, respectively, depending on whether the pitch was relative or absolute), or to recognize a relationship between two notes to be identified. P140 specified: “*I can distinguish a scale, (…) steps, where each step corresponds to a note. And then, when I move downward, my eyes follow the steps in my head”*. Some musicians drew diagrams to explain their visual anchorpoints (Figure 2C): P132 saw patterns represented by large and small boxes respectively for tones and semitones; for P164, when the stimulus was a D sharp, he mentally saw an A and estimated the distance between the two mental reference points. P112, who had absolute pitch, mentally visualized small forms corresponding to the heard notes and drew those forms on a conceptualized keyboard (Figure 2C). She said: *“The B, it’s clear, I see a kind of pike… The position [on the keyboard] of the B flat, for example, the last black key in relation to the white keys … we place it downward, and always in relation to the C scale that is the first scale we learn, this comes before… thus the B flat will close, will protect such C scale from above… One does not find, for the B flat, the sharpness of the B natural… the B natural, it really makes an angle! The B natural, I feel it a little bit sharper, and it’s a shape that would open this way* [she draws a diagram]. *The B natural, to me, opens [the way to] another scale and, at the same time, comes before the C … thus this kind of shape…”*

##### MA8: “View” of a color or matter associated to the note pitch

This MA was used for example to identify single notes, or to refine identification of a note in the presence of an alteration. Some participants referred to colored visions, such as P112 who said: “*I see the E reddish, while the E flat would be more brown… anyway, darker”.* Others saw matter’s effects or deformations. Thereby, P132 described, *“A form (…) which shrinks if the intervals are small and distends when [they] widen. But from a purely visual point of view, a kind of matter (…), like modeling clay.”*

##### MA9: Feeling of a vocal gesture associated to the production of a note

Some participants mentally positioned their phonatory apparatus in accordance with the production of the note. It was the act of provoking a *“mental muscular gesture”* that allowed these musicians to identify the perceived note, similarly to what one would have to actually do to sing it. For example, P132 said: *“I do not think that my phonatory apparatus really positions itself, however, I have the feeling that it does so (…). It’s the same when I imagine a tennis gesture: I have this feeling but my arm does not move.”* This MA was used by musicians who evoked a frequent use of internal mental singing in their musical practice, as for example P144: “*When I hear a very high pitch, for example, then I do retract here* [she showed her throat], *as if I were going to sing it.*”

##### MA10: Feeling of an instrumental gesture associated to the production of a note

Some participants mentally positioned themselves in accordance with the instrumental production of the note: the hand on the violin neck (P165 said: “*I feel the gesture, I feel this specific shifting movement to go from A to F sharp, oh yes, I feel it*”), the gap between the fingers on a piano keyboard (P126: “*I feel the distance that I need to travel with my hands, for example to move from A to E flat”*), etc.

##### MA11: Other miscellaneous feelings specific to a note

This category includes the MAs that were described as any other mental sensations associated to the body: a feeling of openness (P155: *“An enlargement like a rib cage that gradually opens”*); chills or tension (P110: *“I physically feel this tension in the augmented forth, it is located in the upper [part of the] back”*); mental feelings related to thermoception (P101: *“It’s like a thermal sensation”*); a nociception (P132: *“Sometimes, when I listen to singers whose vocal range extends a lot, I almost feel the pain this would have provided me if I would have tried to do the same”).*

#### A Conceptual Framework for the Cognitive Process of Note Recognition

The qualitative analysis outlined so far allowed us to highlight that musicians tap into many different MAs, which can be grouped into eleven categories. All these categories may be used in a mental approach by which a musical note is identified. Such categories can be classified according to the involved sensory modality: auditory, visual or kinesthetic (here, we refer to the capability of *mentally* seeing, *mentally* hearing, *mentally* feeling). During the process of note’s recognition, we hypothesize that MAs are utilized and manipulated in order to create efficient MRs of notes, i.e., a mental image which come to the mind at the stage of recognizing a note or chord and providing an explanation for the perceived external signal. Indeed, the mental processes described by the participants explicitly referred to one of these modalities by means of words (verbs, substantives, etc.) that may be related to each case: *I hear, I see, I feel; an inner voice, a drawing, a chill; etc.* Eventually, it was through MRs that musicians fulfilled the task by communicating the name of the note.

The mental processes described by the participants seemed to be organized sequentially, making it possible to propose a conceptual framework consisting of several stages, beginning with the detection of the stimulus by the inner ear and ending with a verbal output corresponding to the name of the note(s) (Table 3). The starting point was the sensory stimulus of different complexity and with well-established physical nature and location in the external space, here the musical note(s). **Step 1 – Sensory detection** is the picking up of the external signal by any sensory organ, here the inner ear, and following by transmission to brain entities. **Step 2 – Selection of mental anchorpoints** is the stage when fragments of thought emerge, scaling according to the higher or lower complexity of the external stimulus and to the training background of the recipient. As anticipated in the previous section, we called these elements of thought “mental anchorpoints”. MAs come into consciousness during introspection, and contribute to the interpretation of the external signal; depending on the context (e.g., isolated note versus notes in a chord), the same signal can trigger the selection of different anchorpoints. In our formulation, MAs correspond to entities which store memories of previous perceptions or actions which can arise from multiple sensory modalities. **Step 3 – Mental integration** is the process of connecting one or more MAs in order to select, endorse and/or support a (possibly conscious) MR of the perceived signal. This corresponds to the stage of aggregation and/or validation of MAs. Many different operations are here performed, such as superposition or blending of anchorpoints, with different degrees of proximity and precision. This represents a complex cognitive task, which requires combining several levels of intervention and different intentional aims. What happens here is a sort of forging an amalgam, in order to get a meaningful conclusion. Depending on the difficulty of the task and the background/expertise of the participant, one could observe different levels of access to consciousness. Such integration is illustrated in Table 3 for participant P126. In this case, it was possible to progressively identify the note, as well as to validate such identification, by combining information from each of the following anchorpoints: (a) inner hearing of the syllable *“i”*, which put on the track of a probable E (in French, *mi*); (b) identification of the consonant *“m”* preceding the vowel *“i”* (a specific process described by P126 himself: “*The consonant comes to me”*); (c) instantaneous recognition of the triton (augmented fourth interval): as a reference A was provided by the experimenter, P126 almost instantly refined his previous answer, consisting of the syllable *“mi”* (steps a and b), and inferred that the note was actually an E flat; (d) final confirmation by association of the previous three steps with the mental gestures related to *“the physical sensation of [my own] phonatory apparatus”* and *“the gap between the fingers.”* The interweaving of MAs, echoes of previous perceptual experiences, and building blocks of emotional or aesthetic valence appeared to be an important expression of the mental strategies implemented during notes’ identification. However, the role of such building blocks, alongside MAs and MRs, remains to be clarified. **Step 4 – Validation of mental representations** is the stage of ascribing a meaning to the perceived stimulus, i.e., a mental image (in the sense of a MR, therefore not necessarily a visual image) comes to the mind during conceptual grasping, more or less consciously, to provide an explanation for the perceived external signal. As mentioned above, we called these inner images “mental representations.” MRs are personal and may differ from one individual to another. They are not usually communicated as such, except during introspection. **Step 5 – Verbalization** is the final stage indicating successful identification of the stimulus by verbalization through standard terminology. Neither the nature of the underlying MR nor the associated MA(s) are hereby communicated; however, the recognition of the perceived object is completely established.

**TABLE 3:** Conceptual framework for mental anchorpoints.

Step	Description of the process	Examples from P126’s interview
Stimulus	A note is played on the piano; in case, it might be preceded by a reference A (on demand).	

(1) Sensory detection	The participant hears the notes.	

(2) Selection of mental anchorpoints	Several mental anchorpoints are selected: • An inner voice “pronounces” one or more syllables • An inner vision of a part of an instrument emerges • A mental vocal gesture is felt • A mental instrumental gesture is felt • An interval is recognized	• *I heard “iiii”* *• I see a keyboard* *• My physical feeling (…) is (…) associated to a vocal physical feeling* *• I feel the gap between the fingers in my hand (…). I would use fifth and second fingering* *• I suddenly hear the triton*

(3) Mental integration	It’s the process of combining partial information from several anchorpoints. For instance: • The ending syllable, e.g., *i* • The interval’s signature • Identification of a keyboard portion • Various mental gestures (vocal, physical, etc.)	• *When I hear the notes, I hear vowels, thus I suppose it is an E (mi)… Until I get another hint, (…) it is difficult…* • *This interval signature is very familiar to me* *• I identify the middle part of the keyboard* *• The physical feeling that I perceive in my phonatory apparatus in association to a given interval corresponds to the physical feeling I perceive in my hand, arm or elbow*

(4) Validation of mental representation	When mental anchorpoints are amalgamated to each other, a conceptual grasp is achieved. Two strategies may optionally overlap: one direct, the other one indirect. This allows to refine, confirm and stabilize a non-ambiguous mental representation: • Direct strategy: the name of the note (among the seven diatonic possibilities) and its possible alteration (flat, sharp or natural) • Indirect strategy: amalgamation of mental anchorpoints to (i) recognize interval signatures; (ii) measure the distance between two points	• *I better refine the vowel. Here, clearly, I hear a consonant coming out, I even know in which tonality I stand* • *I hear the interval, however, I also physically feel the distance that I should make with my hands, for example, to go from A to E flat. “Geographically” speaking, I perfectly “see” how it works, even included the gap required to obtain this on the keyboard*

(5) Verbalization	The answer is provided	• *It’s an E flat*

### Quantitative Analysis

In order to assess this conceptual framework, i.e., to specifically precise the relationship between MAs and MRs, we undertook a more systematic approach consisting in a quantitative analysis of the content of all interviews. First, in a pilot experiment with ten musicians, we extracted and quantified any reference to MAs, MRs, and different manipulations of the former and the latter. After validation of this procedure, we extended our quantitative analysis to all interviews (56 in total) to (i) quantify the usage of single categories of MAs, (ii) classify the usage of MAs according to gender and expertise, (iii) search and discuss simultaneous and sequential usage of MAs, (iv) look for possible grouping of participants based on sensory modalities, and (v) investigate the possible relationships between different anchorpoints. These analyses are presented in the next subsection.

#### Pilot Study on a Set of 10 Musicians

First, we selected a sample of 10 musicians based on the diversity of their profiles and the richness of their interviews (see Table 1). We systematically extracted textual elements referring to any of the three key elements of our model: (i) MAs, assigning each of them to one of the above-defined categories MA1 to MA11 (step 2); (ii) manipulation of MAs for building up MRs (step 3); (iii) MRs associated to the three sensory modalities – auditory, visual and kinesthetic (step 4). The text encoding was performed independently by two of us (AL and PL), followed by a double check of all the occurrences and a two-man systematic validation. A Cohen’s kappa test was performed to check the consistency between the two independent annotators, indicating a substantial agreement between them for eight participants (coherence indices between the two annotators between 0.524 and 0.829; see Table 4). In addition, the seek for convergence in the assignments of occurrences by the two annotators allowed us to specify the demarcation points between different MAs, especially between MA2 and MA4. The low Cohen’s kappa indices for the two participants P101 and P123 can be commented as follows. P101 used several MAs of a different kind in combination, therefore it wasn’t straightforward to make some text elements exactly correspond to a unique category of anchorpoints (initial Cohen’s kappa index between the two annotators of 0.420). P123 described in a very precise way the concept of mental “spatialization” when “hearing” the name of the notes. The two annotators initially interpreted such spatialization in association with the visual space, before reaching the conclusion that this musician was rather referring to the spatialization of the sound source, without any reference to the visual field. This produced a discrepancy, observed for many occurrences, between the initial/individual and final/joint assignments (Cohen’s kappa indices of 0.314 and 0.346, respectively for AL and PL) as opposed to the coherence observed between the two initial individual assignments (Cohen’s kappa index of 0.661).

**TABLE 4:** Validation of the qualitative method used to assign the occurrences of mental anchorpoints and mental representations (Cohen’s kappa test).

Participant	Mental			Mental		
	anchorpoints (MA)			representations (MR)		
	R_1_ – R_2_	R_1_ – R_12_	R_2_ – R_12_	R_1_ – R_2_	R_1_ – R_12_	R_2_ – R_12_
P101	0.420	0.657	0.734	1	1	1
P110	0.655	0.868	0.770	0.443	0.780	0.620
P112	0.781	0.914	0.847	0.671	0.928	0.744
P123	0.661	0.314	0.346	0.277	1	0.277
P126	0.804	0.967	0.835	0.810	0.865	0.873
P132	0.607	0.759	0.824	0.794	0.794	1
P136	0.524	1	0.524	1	1	1
P144	0.530	0.845	0.653	0.465	0.867	0.596
P148	0.685	0.875	0.723	1	1	1
P163	0.829	0.928	0.865	0.769	0.769	1
All	0.723	0.819	0.753	0.591	0.913	0.663

*R_1_, annotator AL; *R*_2_, annotator PL; *R*_12_, final annotation after discussion and agreement between AL and PL. In this test, a score of 1 indicates a perfect match, a score between 0.81 and 1 corresponds to an almost perfect agreement, a score between 0.61 and 0.80 indicates a substantial agreement, a score between 0.41 and 0.60 corresponds to a moderate agreement, a score between 0.21 and 0.40 indicates none to slight agreement, and a score below 0.21 indicates a result consistent with a random annotation (McHugh, 2012).*

Our analysis returned nearly 1000 occurrences for MAs and more than 450 occurrences for MRs (Table 5). The quantitative study then focused on steps 2 and 4 of the proposed conceptual framework. Table 5A reports the number of occurrences of MAs in each of the ten interviews on the pilot group. Each musician used several types of anchorpoints, but some of them were privileged such as MA1 (“Hearing” the name or a part of the name of a note). Some anchorpoints (e.g., MA2: Recognition of formally learned intervals or sound patterns that combine several notes) were used as primary supports by some musicians and as marginal supports by others. Finally, some MAs were rarely used, or selected, by only a limited number of participants. These were the cases for MA6 (“View” of a musical instrument or “view” of the body position required to play a note), or MA7 (“View” of individually contrived specific patterns that correspond to a note or to a group of notes). In Table 5B, we report the number of occurrences of MRs in each of the ten interviews on the pilot group. Three major sensory modalities are here represented: auditory, visual and kinesthetic. Auditory-type MRs were very widely used; however, visual and kinesthetic representations were also evoked by a large number of musicians.

**TABLE 5:** Occurrences of textual elements extracted from the interviews, pilot group of 10 participants.

(A)												

Participant	MA1	MA2	MA3	MA4	MA5	MA6	MA7	MA8	MA9	MA10	MA11	Total
P101		8	20	15				10	3		13	69
P110		34					14	6	6		58	118
P112	41	1		22	1	8	17	10	5	16	6	127
P123	115	1		14					9	8	5	152
P126	60	6			2	13			26	22	1	130
P132	3	6	7			3	59	6	2		1	87
P136	1	22		9	1	1				1		35
P144	50	5							13		18	86
P148	48	40		1		21				1		111
P163	29	5			8	4	2					48
Total	347	128	27	61	12	50	92	32	64	48	102	963

**(B)**												

**Participant**	**Auditory**				**Visual**			**Kinesthetic**				**Total**

P101	28											28
P110	24				15			18				57
P112	25				22			8				55
P123	91				4			17				112
P126	23				5			15				43
P132	2				20			5				27
P136	33				1							34
P144	30							20				50
P148	24				3							27
P163	19				4							23
Total	299				74			83				456

*(A) Occurrences for each category of mental anchorpoint (see Table 2 for definition). (B) Occurrences for mental representations, according to the primary sensory modality.*

**FIGURE 3:**
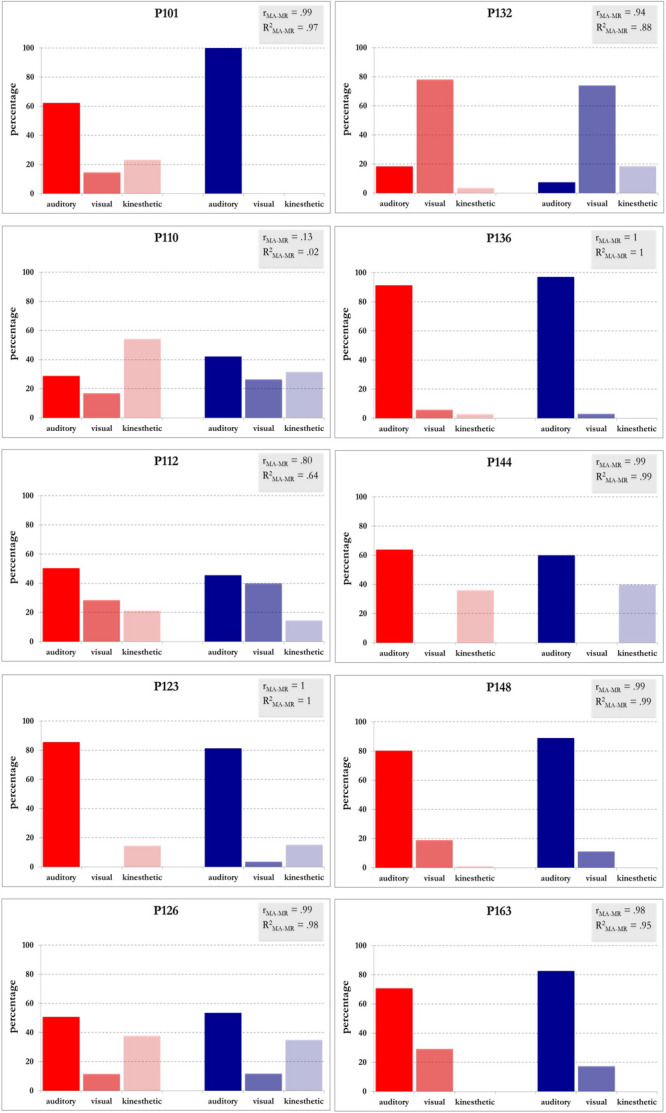
Comparison between occurrences for mental anchorpoints and mental representations, pilot group of ten participants. In red, occurrences of mental anchorpoints (MA) grouped by sensory modality. MA1–MA4, auditory; MA5–MA8, visual; MA9–MA11, kinesthetic. In blue, occurrences of mental representations (MR) of auditory, visual and kinesthetic modality. *r*_*MA–MR*_ and *R*^2^ indicate Pearson correlations and coefficients of determination between occurrences of MA and MR, respectively. The coefficients of determination *R*^2^ illustrate, case by case, the proportion of the variance in the three modalities of mental representations (dependent variables) that is predictable from the three modalities of mental anchorpoints (independent variables).

Each MA mainly relates to a specific sensory modality: namely, MA1 to MA4 relate to the auditory modality, MA5 to MA8 to the visual modality and MA9 to MA11 to the kinesthetic modality. This resulted in three main groups of anchorpoints. For each musician, it was therefore possible to identify the sensory modality related to each group of anchorpoints and to compare it with the number of occurrences of the mental representations of the same modality. This comparison is shown in Figure 3. Note that the histograms representing the occurrences of MAs and MRs are extremely similar for most musicians. The correlation coefficients between these two data sets are close to 1, with the exception of P110. This participant regularly used a very special MA, MA11, indicating a body feeling specific to each music interval. Her interview was very detailed about this aspect, leading to numerous occurrences for MA11. Nevertheless, her MRs of kinesthetic modality was not discussed so in depth, leading to an underrepresentation of this modality.

The correlation between the typology of MAs and the sensory modality related to MRs suggested a functional relationship such as a mental integration or fusion of MAs for building up MRs (step3). We extracted the text elements corresponding to this step. The vocabulary was extremely diversified from one musician to another (results not shown). We detected associations, fusions, comparisons and other manipulations of MAs. This could both reflect the diversity in the usage of MAs and be a consequence of different degrees of awareness in the process of introspection. To investigate this aspect would require a detailed separate study, and it has not been further examined hereby.

#### Complete Analysis on the Whole Group of 67 Musicians: Selection of MAs and Effect of Expertise

In a next step, we counted the occurrences of MAs for each of the 11 categories in the remaining interviews. In 11 of them, it was not possible to properly extract the anchorpoints, so those participants were excluded from further analyses. The first case was a student, too young to satisfactorily complete the recognition task (P137, 6 years old). Other four students, all guitarists, could identify the notes only by referring to the position on the guitar neck and were not able to name them (P106, P115, P141, and P159). For three professional musicians (P146, P147, and P152), the interviews were conducted without any auditory test, so their mental descriptions were too generic and limited to discourses on how they heard the notes. Note that one of these musicians (P147), who played an African instrument, the balafon, was not used to practice pitch recognition on his instrument; he was rather used to capture, according to the musical context, a semantic meaning. This is an example of one of the limitations of our study, being confined to the context of Western classical music. Finally, for three professional musicians (P125, P127, and P134), the number of occurrences of anchorpoints identified in the text was too low (less than ten in total) to be taken into account.

The identification of MAs was thus carried out for 46 additional participants. For this group, assignments of occurrences for each category were jointly provided by two of the authors (AL and PL). A Kolmogorov–Smirnov (KS) test was performed, in order to check whether the results were consistent with those obtained for the pilot group (Table 6). For ten of the eleven categories of MA, the KS test returned a positive answer: the null hypothesis, stating that the distribution of MAs between the various categories in the pilot group was the same as for the rest of the musicians, was correct at a 95% confidence level. In the case of MA11 (feelings specific to a note, different from vocal or instrumental gestures), the results for the two groups were not consistent with each other in that a much higher percentage of participants (70% i.e., 7/10) in the pilot group mentioned MA11; in contrast, only six new participants (13% i.e., 6/46) referred to such body sensations. This may be a consequence of the wide diversity in the profile of subjects selected in the initial pilot study. The number of occurrences in each category of MA for all the 56 participants is reported in Table 7.

**TABLE 6:** Kolmogorov–Smirnov test.

*H*_0_: the distribution of mental anchorpoints among the 11 categories in the pilot group is the same as for the rest of the musicians		

MA	*p*	*H*_0_ is correct (*CL* = 95%)
MA1	0.469	Yes
MA2	0.347	Yes
MA3	0.451	Yes
MA4	0.136	Yes
MA5	0.316	Yes
MA6	0.883	Yes
MA7	0.795	Yes
MA8	0.190	Yes
MA9	0.114	Yes
MA10	0.715	Yes
MA11	0.010	No

*Consistence of the methods used to assign occurrences of mental anchorpoints to the two groups: pilot group of 10 participants and remaining 46 participants (assumption of independent samples). *H*_0_, null hypothesis; *p*, probability to satisfy *H*_0_; *CL*, confidence level.*

**TABLE 7:** Number of occurrences of mental anchorpoints, complete group of 56 musicians.

Participant	MA1	MA2	MA3	MA4	MA5	MA6	MA7	MA8	MA9	MA10	MA11	Total
P101		8	20	15				10	3		13	69
P102		2	5			21				19		47
P103			10	5	24		1					40
P104			6	9	1					2		18
P105	5	6	2	3	14	2			14	9		55
P107		6		7		5						18
P108	1		6	7		7			2			23
P109	18	10	29	3	21				3	4		88
P110		34					14	6	6		58	118
P111	6	4	5	8	1	6				1		31
P112	41	1		22	1	8	17	10	5	16	6	127
P113					13					2		15
P114		2		40		3				12		57
P116		1	27			3			6			37
P117	14			7								21
P118				4		10				2		16
P119	12	26		1		2						41
P120	8	6	1	3								18
P121		10				9				4	3	26
P122	38	3		5						9		55
P123	115	1		14					9	8	5	152
P124	10	6		7	14	3	2			10		52
P126	60	6			2	13			26	22	1	130
P128	4	3		36		42	6			4		95
P129		1	14	5	8	4	14			4		50
P130	61			11	2		3			1	1	79
P131		12		41		5			3	11	1	73
P132	3	6	7			3	59	6	2		1	87
P133	3	36		8		10			7	17	5	86
P135		4	13	3	1	23			7			51
P136	1	22		9	1	1				1		35
P138	22	1		21								44
P139	26		4	13					8			51
P140	24	10	30	23	6		14			3		110
P142	1	19		2					2	1		25
P143			9		18							27
P144	50	5							13		18	86
P145	7	3	8		7	1	12		4			42
P148	48	40		1		21				1		111
P149	15	6	7	16								44
P150	8	12	7	2	15					3		47
P151	49		1	1		7				3		61
P153	25			5	9	1						40
P154	20				1					1		22
P155			7			1	32	1	1	5	4	51
P156		1	6		14	3				1		25
P157	13		1	6	7		3		9	1		40
P158		8	5	3	10		1					27
P160	8	12	1	4	17	1				11		54
P161	31	12		19	9	5				5		81
P162	26	2		14								42
P163	29	5			8	4	2					48
P164		42		10		16	28	18		1		115
P165	13	24		8		13	2		4	17	1	82
P166	19	2		4	1		14			2		42
P167	10			9	7	13			1			40
Total	844	420	231	434	232	266	224	51	135	213	117	3167

*The total number of occurrences for each musician is indicated on the far right column. The total number of occurrences for each mental anchorpoint is indicated at the bottom line.*

The range for such entries is quite broad (between 0 and 115). In some cases an entry might be overrepresented just because the interview focused more deeply on a given MA, without this necessarily meaning that such anchorpoint was more important than the others (see the case of P110, discussed above). To standardize these results, only the entries with at least 10% of the total number of occurrences for a given participant were counted. For the interviews with a low number of occurrences (less than 30), only the entries with at least three occurrences were taken into account. Conversely, for very rich interviews (more than 100 occurrences), we retained all the entries with at least ten occurrences. Such an analysis returned, for each musician, a signature for the usage of her/his preferred MAs, with the exclusion of those less used. This allowed us to convert the rough set of occurrences displayed in Table 7 into a binary set of 0 and 1, by converting representative (i.e., retained) entries into 1 and non-representative (i.e., a few or null) entries into 0, as indicated in Table 8.

**TABLE 8:** Binary signatures for mental anchorpoints, complete group of 56 musicians.

Participant	MA1	MA2	MA3	MA4	MA5	MA6	MA7	MA8	MA9	MA10	MA11	Total
P101	0	1	1	1	0	0	0	1	0	0	1	5
P102	0	0	1	0	0	1	0	0	0	1	0	3
P103	0	0	1	1	1	0	0	0	0	0	0	3
P104	0	0	1	1	0	0	0	0	0	0	0	2
P105	1	1	0	0	1	0	0	0	1	1	0	5
P107	0	1	0	1	0	1	0	0	0	0	0	3
P108	0	0	1	1	0	1	0	0	0	0	0	3
P109	1	1	1	0	1	0	0	0	0	0	0	4
P110	0	1	0	0	0	0	1	0	0	0	1	3
P111	1	1	1	1	0	1	0	0	0	0	0	5
P112	1	0	0	1	0	0	1	1	0	1	0	5
P113	0	0	0	0	1	0	0	0	0	0	0	1
P114	0	0	0	1	0	0	0	0	0	1	0	2
P116	0	0	1	0	0	0	0	0	1	0	0	2
P117	1	0	0	1	0	0	0	0	0	0	0	2
P118	0	0	0	1	0	1	0	0	0	0	0	2
P119	1	1	0	0	0	0	0	0	0	0	0	2
P120	1	1	0	1	0	0	0	0	0	0	0	3
P121	0	1	0	0	0	1	0	0	0	1	1	4
P122	1	0	0	1	0	0	0	0	0	1	0	3
P123	1	0	0	1	0	0	0	0	0	0	0	2
P124	1	1	0	1	1	0	0	0	0	1	0	5
P126	1	0	0	0	0	1	0	0	1	1	0	4
P128	0	0	0	1	0	1	0	0	0	0	0	2
P129	0	0	1	1	1	0	1	0	0	0	0	4
P130	1	0	0	1	0	0	0	0	0	0	0	2
P131	0	1	0	1	0	0	0	0	0	1	0	3
P132	0	0	0	0	0	0	1	0	0	0	0	1
P133	0	1	0	0	0	1	0	0	0	1	0	3
P135	0	0	1	0	0	1	0	0	1	0	0	3
P136	0	1	0	1	0	0	0	0	0	0	0	2
P138	1	0	0	1	0	0	0	0	0	0	0	2
P139	1	0	0	1	0	0	0	0	1	0	0	3
P140	1	1	1	1	0	0	1	0	0	0	0	5
P142	0	1	0	0	0	0	0	0	0	0	0	1
P143	0	0	1	0	1	0	0	0	0	0	0	2
P144	1	0	0	0	0	0	0	0	1	0	1	3
P145	1	0	1	0	1	0	1	0	1	0	0	5
P148	1	1	0	0	0	1	0	0	0	0	0	3
P149	1	1	1	1	0	0	0	0	0	0	0	4
P150	1	1	1	0	1	0	0	0	0	0	0	4
P151	1	0	0	0	0	1	0	0	0	0	0	2
P153	1	0	0	1	1	0	0	0	0	0	0	3
P154	1	0	0	0	0	0	0	0	0	0	0	1
P155	0	0	1	0	0	0	1	0	0	1	0	3
P156	0	0	1	0	1	1	0	0	0	0	0	3
P157	1	0	0	1	1	0	0	0	1	0	0	4
P158	0	1	1	1	1	0	0	0	0	0	0	4
P160	1	1	0	0	1	0	0	0	0	1	0	4
P161	1	1	0	1	1	0	0	0	0	0	0	4
P162	1	0	0	1	0	0	0	0	0	0	0	2
P163	1	1	0	0	1	0	0	0	0	0	0	3
P164	0	1	0	1	0	1	1	1	0	0	0	5
P165	1	1	0	1	0	1	0	0	0	1	0	5
P166	1	0	0	1	0	0	1	0	0	0	0	3
P167	1	0	0	1	1	1	0	0	0	0	0	4
Total	31	24	18	32	17	16	9	3	8	13	4	175

*1, usage of the mental anchorpoint; 0, non-usage of the mental anchorpoint. The number of different categories of mental anchorpoints used by each subject is indicated on the far right column. The number of subjects using a given category of mental anchorpoint is indicated at the bottom line of the table.*

Following this procedure, we found that six categories were more widely used by at least sixteen musicians: MA1 to MA6. Two of these were most represented: MA1, “Hearing” the name or part of the name of a note, 31 musicians; and MA4, note recognition by association of pitch with specific auditory hues, or the notion of “timbre-pitch,” 32 musicians. Three categories were seldom selected (MA7, MA9, and MA10, by respectively 9, 8, and 13 musicians) and two categories were very rarely used (MA8 and MA11, respectively by 3 and 4 musicians). It can be noted that the four auditory categories, MA1–MA4, were the most selected. On average, participants selected three categories; some of them mentioned only one category, whereas others mentioned up to five categories.

In addition, quite surprisingly for a task of note identification, some professional musicians (P101, P112, P135, and P161) ascribed different kinds of emotional or aesthetic valence to the note; this noticeably impacted the process of recognition and should be considered as a complementary feature. For example, P112 said: “*The note B is weak, fragile, it becomes a bit endearing and one feels it small*,” while P161 stated: “*G major reminded me of a [musical] piece of prime importance, which is the great Fantasy for organ by [Johann Sebastian] Bach (…) I was sent back to the womb.*” These observations would require further investigation.

The very varied use of MAs prompted us to take a closer look at the participant’s status (gender and expertise, see Table 9). Comparison between students and professionals showed that the search for note pitches by the use of rising or descending scales (MA3), as well as “view” of the note inside the stave, or “view” of the name of the note (MA5), were primarily used by students, whereas recognition of formally learned intervals or sound patterns that combine several notes (MA2) or feeling of an instrumental gesture associated to the production of a note (MA10) were more used by professional musicians (*p*-values < 0.005 and equal to 0.029, 0.011 and 0.061 for MA3, MA5, MA2, and MA10, respectively). The low number of female musicians (4) in the professional group (22) did not allow us to analyze the effect of gender. By contrast, comparison between male and female students made it possible to highlight that categories MA5 and MA9 (feeling of a vocal gesture associated to the production of a note) were primarily used by female students (*p*-values of 0.002 and 0.006 for MA5 and M9, respectively). Moreover, in the male group, MA3 was mainly selected by students (8 students versus 1 professional musician, *p*-value of 0.018), whereas MA2 was mainly used by professional musicians (11 professional musicians versus 5 students, *p*-value of 0.044). The seeming preferred usage of MA10 by male professional musicians (7 professional musicians versus 3 students), was not statistically significant (*p*-values of 0.137).

**TABLE 9:** Usage of the different mental anchorpoints by musicians, according to their gender and expertise.

Sample size	Group	MA1	MA2	MA3	MA4	MA5	MA6	MA7	MA8	MA9	MA10	MA11
56	All	31	24	18	32	17	16	9	3	8	13	4
34	Students	19	10	17	16	14	8	6	0	6	5	1
22	Professionals	12	14	1	16	3	8	3	3	2	8	3
16	Female students	8	5	9	6	11	3	2	0	6	2	1
18	Male students	11	5	8	10	3	5	4	0	0	3	0
18	Male professionals	10	11	1	14	2	7	1	2	2	7	2

*The total number of participants per group is indicated on the left.*

Inspired by the strong correlation between MAs and MRs on the pilot group (cf. Figure 3), we clustered the 56 participants according to their preference for the different sensory modalities (Table 10). Following our classification of MAs into three categories, i.e., auditory (MA1–MA4), visual (MA5–MA8) and kinesthetic modalities (MA9–MA11), participants were clustered into five groups: strictly auditory; strictly visual; auditory and visual; auditory and kinesthetic; auditory, visual and kinesthetic. With the exception of the two students (P113 and P132) belonging to the strictly visual group, participants were clustered according to sensory modalities that always included the auditory dimension: strictly auditory, 12 participants; auditory and visual, 21 participants; auditory and kinesthetic, 6 participants; auditory, visual and kinesthetic, 15 participants. Student P113 used only one single anchorpoint, MA5 (“view” of the note inside the stave). Note that MA5 was generally used by students. This suggests that P113 might still own a weak strategy for pitch recognition. Student P132 selected MA7, using patterns illustrating small parts of the keyboard (Figure 2C). His MA corresponded to a schematic representation of the intervals of major and minor second. However, in his description, a few other MAs of auditory type, such as the search for note pitches by the use of rising or descending scales (MA3) were mentioned (Table 7). Such auditory anchorpoints were clearly present, although rarely quoted *“I hear the notes following one another in my head, (…) I go up an interval, (…) as if I were singing.”* These finding positioned him close to the auditory-visual group.

**TABLE 10:** Grouping of participants based on references to sensory modalities.

Sensory modality	Participant	Expertise	Gender	Absolute or relative pitch	MA1	MA2	MA3	MA4	MA5	MA6	MA7	MA8	MA9	MA10	MA11
Auditory	P154	STU	M	ND	1										
	P119	STU	M	ND	1	1									
	P117*	STU	M	ND	1			1							
	P123*	PRO	M	abs	1			1							
	P130*	PRO	M	abs/rel	1			1							
	P138*	PRO	M	abs	1			1							
	P162*	STU	M	ND	1			1							
	P120	PRO	M	ND	1	1		1							
	P149	STU	M	abs	1	1	1	1							
	P142	STU	M	rel		1									
	P136	PRO	M	rel		1		1							
	P104	STU	M	ND			1	1							

Auditory and visual	P151	STU	M	ND	1					1					
	P163	PRO	F	abs	1	1			1						
	P148	PRO	M	rel	1	1				1					
	P153	STU	F	ND	1			1	1						
	P166	STU	M	ND	1			1			1				
	P167	STU	M	ND	1			1	1	1					
	P109*	STU	F	ND	1	1	1		1						
	P150*	STU	M	ND	1	1	1		1						
	P161	PRO	M	abs	1	1		1	1						
	P111	STU	M	ND	1	1	1	1		1					
	P140	STU	F	ND	1	1	1	1			1				
	P107	PRO	F	rel		1		1		1					
	P164	PRO	M	abs		1		1		1	1	1			
	P158	STU	F	ND		1	1	1	1						
	P143	STU	F	ND			1		1						
	P156	STU	F	ND			1		1	1					
	P103	STU	F	ND			1	1	1						
	P129	STU	M	ND			1	1	1		1				
	P108	STU	M	abs			1	1		1					
	P118*	STU	F	ND				1		1					
	P128*	PRO	M	abs				1		1					

Auditory and kinesthetic	P144	STU	F	rel	1								1		1
	P139	PRO	M	abs	1			1					1		
	P122	STU	M	ND	1			1						1	
	P131	PRO	M	abs/rel		1		1						1	
	P116	STU	F	ND			1						1		
	P114	PRO	M	abs				1						1	

Auditory, visual and kinesthetic	P126	PRO	M	abs	1					1			1	1	
	P105	STU	F	ND	1	1			1				1	1	
	P160	STU	F	ND	1	1			1					1	
	P145	STU	F	ND	1		1		1		1		1		
	P157	STU	F	ND	1			1	1				1		
	P112	PRO	F	abs	1			1			1	1		1	
	P124	PRO	M	rel	1	1		1	1					1	
	P165	PRO	M	abs	1	1		1		1				1	
	P133	PRO	M	rel		1				1				1	
	P121	PRO	M	rel		1				1				1	1
	P110	PRO	F	rel		1					1				1
	P101	PRO	M	rel		1	1	1				1			1
	P135	STU	F	ND			1			1			1		
	P102	STU	M	ND			1			1				1	
	P155	STU	M	ND			1				1			1	

Visual	P113	STU	F	ND					1						
	P132	STU	M	ND							1				

MA1–MA4, MA5–MA8, and MA9–MA11 categories refer to auditory, visual and kinesthetic modalities, respectively. The five identified groups are indicated on the left column. Expertise (student – STU or professional – PRO), gender (M or F) and type of hearing (absolute pitch – abs, relative pitch – rel, not determined – ND) are displayed in columns from 3 to 5. Participants whose signatures were identical (5 subjects in the strictly auditory group, and 2 pairs of subjects in the auditory-visual group) are indicated with an asterisk (*). Note that two participants reported to have absolute or relative pitch depending on the context (P130 and P131).

The analysis by groups of distinct sensory modality led us to several observations: first, with the exception of the two students discussed above, all participants used auditory mental supports for note’s identification. Second, the majority of participants (36 participants out of 56, both students and professional musicians) used visual modality in association with auditory modality for their multimodal representations of notes. Seven out of 11 categories of MAs (MA5–MA11) implied a non-auditory sensory mental modality. MA1, which corresponds to mentally hearing the name of the note, also provides an example of a sensory transmodal strategy, in this case relating sound and language – something that might be interpreted as a kind of synesthesia (Chun and Hupé, 2016). More specifically, MAs and MRs whose descriptions by the 56 participants implied a strictly auditory modality are in the minority: only 12 musicians adopted MRs of this kind, with 9 out of them selecting the anchorpoint MA1 (Table 10). Out of 175 entries, only 74 related to MA2, MA3, and MA4, i.e., anchorpoints of strictly auditory sensory mental modality (Table 9). Moreover, all groups presented a balanced gender distribution, with the exception of the strictly auditory group that was exclusively composed by males. Such group comprised seven male students; by comparison, the auditory-kinesthetic-visual group, comprised five girls and two boys.

As a further step, we looked more closely at a possible relationship between absolute/relative pitch and the use of specific MAs, as well as the sensory modality of MRs (Table 10). Note that although all professional musicians, with the exception of a percussionist (P120), were able to qualify the nature of their hearing, only some student could do that. All the participants (*n* = 15) who declared to have absolute pitch, except two, were able to identify a specific auditory signature (or hue) for each note (MA4). Similarly, all the participants (*n* = 12) who declared to have relative pitch, except two, were able to identify the note pitch from a typical interval signature (MA2). Indeed, it can be noted that participants, whether they had absolute or relative pitch, belonged to any of the groups related to different sensory modalities (Table 10). Therefore, while the use of certain mental anchors is related more specifically to an absolute or a relative pitch (e.g., MA4 for absolute pitch and MA2 for relative pitch), the typology of MRs, as reported by the multimodal sensory groups (auditory, auditory-visual, etc.), does not distinguish between these two hearing abilities.

**FIGURE 4:**
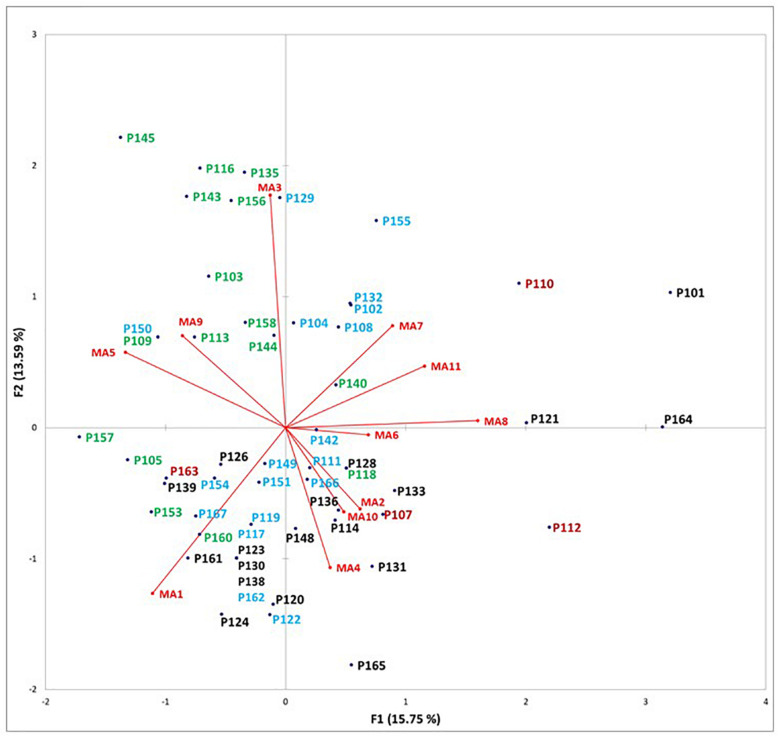
Principal component analysis on mental anchorpoints. Profiles of the main identified functional modalities, in terms of the usage of mental anchorpoints (red vectors) by participants (points). Correlation biplot for the two most significant new variables is provided: Female students, 16 participants, green; Male students, 18 participants, light blue; Female professionals, 4 participants, brown; Male professionals, 18 participants, black.

Finally, we asked ourselves whether the detected anchorpoints, although expressing a rich expression of individual diversities, might conceal a smaller number of cognitive features accounting for a more limited set of mental strategies for pitch recognition. To answer this question, we performed a principal component analysis on all the 11 envisaged categories of MAs, with the aim of reducing the number of variables (the anchorpoints). It was not possible to substantially reduce the number of variables to explain the overall variance, as explaining the 74% of the total variance would require at least six new variables, that is more than 50% of the initial number of variables (i.e., the 11 categories of anchorpoints; Table 11). By considering the two more influential new variables, only the 29% of the total explained variance is accounted for. One may note that, according to these two dimensions, participants were differently distributed depending on their expertise (students or professional musicians) and gender (males or females) (Figure 4). A miscellany of MA3, MA5, and MA9 (use of rising or descending scales, “view” of the note inside the stave, or feeling of a vocal gesture) was mostly used by students, particularly by the girls. This distribution suggests that learning in the conservatoires may trigger, differently according to the gender, usage of specific MAs.

**TABLE 11:** Principal component analyses (PCA) on the distribution of mental anchorpoints for all participants.

	F1	F2	F3	F4	F5	F6	F7	F8	F9	F10	F11
Explained variance (%)	15.75	13.59	13.15	11.68	11.03	8.74	6.92	6.24	5.24	3.91	3.75
Explained variance (cumulative proportion)	15.75	29.34	42.49	54.17	65.20	73.95	80.86	87.11	92.34	96.25	100

*Explained variance (%): for each new dimension Fi, percentage of data that is explained by Fi; Explained variance (cumulative proportion): for each new dimension Fi, total variance in the data that is accounted by a linear combination of Fj, j = 0,i.*

As a whole, principal component analysis did not return new prototypic mental strategies for pitch recognition. On the contrary, the diversity of the initially described individual strategies using an idiosyncratic combination of MAs was re-emphasized.

## Discussion

This study questioned the topic of the recognition of note pitches by students and expert musicians. Our aim was to decipher mental strategies used by participants to perform this task. We adopted a phenomenological approach combining qualitative analyses of interviews based on introspection with quantitative statistical analyses carried on the resulting categorization of mental descriptors.

In a first stage, we identified the mental elements which made it possible for musicians to identify and name a note pitch. We called such elements mental anchorpoints. We found that MAs can be of different kind; they can be classified according to their relationship with some of the main sensory modalities, i.e., auditory, visual and kinesthetic. MAs appear to be mostly non-epistemic, since they only rarely capture, if considered in isolation, a well-established conceptual content. It is by their aggregation and association with the intentional purposes of the individuals that activation and stabilization of a conveyable mental representation can be bolstered. We proposed that MAs could act at the same time as vehicles and as elements of thought. Our results indicate the need of making distinctions between MAs and MRs – intended as inner images which come to the mind, more or less consciously, to provide explanation and ascribe a meaning to the perceived external signal. Identification of so many different MAs and their connection with stable MRs of note names underline the strong idiosyncratic connotation of this perceptual process. We observed that certain categories of anchorpoints were preferred by students, while rarely selected by professional musicians. Our detailed analyses on the students’ signatures also showed that male and female students are characterized by different profiles suggesting that they may have adopted different learning attitudes concerning, for example, the time spent in musical dictation, performing or singing.

We acknowledge that a pure phenomenological approach by introspection using interviews to directly address the matter of thought may present some points of weakness (cf. Johansson et al., 2005). We have nevertheless undertaken such an introspective approach (La Garanderie, 1989) in the spirit of the elicitation interview method (Vermersch, 1994). We tried to achieve some rigor, by focusing on the textural and strategic descriptions of mental objects related to a very specific but simple perceptual task, namely the identification of a single or a few notes by musicians. The way we conducted our interviews was similar to recently proposed methods (Vermersch, 2009; Vion-Dury and Mougin, 2018) and in particular to micro-phenomenological interviews, which aim at identifying very precise mental objects in relation to perceptual targets (Petitmengin et al., 2019). Note that our interview method constantly respected the purpose of relating the described mental elements to the proposed task. Intentional aim probably has an influence on the perceptual mental strategies used by interviewees (Petitmengin et al., 2009).

Mental anchorpoints play a central role in note identification by musicians, particularly in relation to individual abilities, such as relative or absolute pitch. For example, MA2 – recognition of formally learned intervals or sound patterns that combine several notes – is frequently associated with relative pitch, while MA4 – note recognition by association of pitch with specific auditory hues – is frequently associated with absolute pitch. The origin of absolute or relative musical listening ability is a subject that has been extensively studied, for example to analyze the influence of the musical context (Eitan et al., 2017) or to identify the neural activation networks responsible for this skill (Wengenroth et al., 2014). The link between absolute pitch and memorization of the notes’ names has already been reported by Takeuchi and Hulse (1993), who observed that individuals with absolute pitch present very different memorization capabilities from one to another. Here, we found that musicians with either absolute or relative pitch abilities may use MA1 – “Hearing” the name or part of the name of a note. We came also to the conclusion that absolute pitch is often… relative: musicians associate, for example, name and pitch only to complex tones and not to pure tones. Although it is quite common to clearly distinguish between such two categories of individuals in the identification of notes, our study shows that these two hearing capabilities are modulated through the implementation of a wide diversity of MAs, allowing important levels of flexibility. Moreover, in both hearing groups, it seems that musicians seek to establish a coherent relationship between MAs and pitches even when the latter are approximately performed: “The ear seeks, through the “inaccuracies” carried by approximations in the pitches, typical musical “beings”” (Francès, 1958; p. 36).

Mental anchorpoints, through their texture and induced strategic contents, provide crucial information on the different ways a sensory stimulus may be conceptualized. In addition, we have observed that the capability to identify a tone pitch does not only depend on the type of MA, but also on musicians’ intentional aims: according with the intention, the conceptual content of a given MA is likely to change. For example, some musicians cannot use MA1, i.e., mental hearing of notes’ names, when a melody contains lyrics, as already reported in the literature (Ihde, 1976). Regards MA2, i.e., recognition of formally learned intervals or sound patterns that combine several notes, the attention might be focused on the intervals separating the notes of a chord or rather on the chord itself as a whole. Similarly, the fact that musicians could either perceive individual notes of a chord or the chord itself as a whole has been previously reported (Stumpf, 1890). MAs can thus trigger perceptive processes of various degrees of complexity with apparently equal efficiency and speed. The texture of MRs is also a concept that has been already addressed in philosophy, for example by Laugier (2003): “A [mental] representation can be a relationship (something that is represented to someone), but [can also act as] a vehicle for the entity that is represented (content, statement, state, perception)” (p. 291). Although MRs are specific to each individual, they nevertheless establish the common ground for communication of perceived signals (Charest and Kriegeskorte, 2015; Stacchi et al., 2019). Interindividual variability in auditory cognition is also studied in specific psychoacoustic experiments (Pelofi et al., 2017). Such experiments could in the future be usefully combined with appropriate phenomenological descriptors of mental strategies.

A significant result of our study is the identification of MAs and MRs of modalities other than auditory. Visual and kinesthetic modalities are well represented. MRs incorporating a visual sensory modality are quite common among musicians (Mongelli et al., 2017) and the kinesthetic modality could prove to be more important than what we found – as also shown by other studies (Brodsky et al., 2008; Godøy and Leman, 2010). In summary, our investigation confirmed the multimodal dimension of any pitch representation. This conclusion is consistent with the hypothesis of a multimodal connotation of any MR, including those related to elementary and apparently one-dimensional stimuli (Nanay, 2018). The simultaneous activation of different sensory modalities by musicians is reminiscent of the phenomenon of synesthesia, a widely addressed subject in cognitive psychology and neuroscience (Gregersen et al., 2013; Bouvet et al., 2014; Chun and Hupé, 2016; Itoh et al., 2017). According to Chiou et al. (2013), sounds elicited consistent visual experiences of colored “geometric objects” located at specific spatial location for a group of synaesthetes. That is, changes in the auditory pitch may alter these visual mental elements in a systematic manner, resembling the cross-modal correspondences we observed hereby. In another study, Loui et al. (2012) asked whether absolute pitch possessors and tone-color synaesthetes might recruit specialized neural mechanisms during music listening. Results supported both shared and distinct neural enhancements. Until now, however, these researches did not converge toward a unique explanation based on precise mechanistic processes. Synaesthesia has been traditionally explained as a purely perceptual phenomenon. However, neuroimaging studies showed that music-color synaesthesia may include some kind of conceptual and semantic inducers. This argues for a move away from a purely “sensory to sensory” explanation (Curwen, 2018). In the same vein, Bragança et al. (2015) proposed the existence of a lower, unconscious degree of synesthesia in non-synaesthetes. Such latent synesthesia, without explicit sensory manifestations, would be functional during the musical experience, where the sensory associations elicited by sound activate memories, images, and emotions. In this regard, our study may suggest new experimental designs to address this point: neuroimaging on subjects who report different anchorpoints and multimodal representations is likely to reveal the involvement of sensory and association areas that are beyond the strict auditory processing network.

In summary, MAs used by musicians for the identification of note pitches illustrate specific individual aptitudes combined with competencies acquired through a learning process which integrates musical theory and practice. In the future, we aim at deepening these observations, by grouping a very large cohort of individuals according to their preference for different categories of MAs, with a special attention for students in the learning stage. In addition, we plan to examine the brain activity of musicians with the same type of hearing (e.g., absolute and/or relative pitch) but using different MAs, and compare their profiles during different tasks for pitch identification - such as recognition of single notes or identification of notes within chords or melodic sequences.

## Data Availability Statement

All datasets generated for this study are included in the article.

## Ethics Statement

Ethical review and formal approval by a committee was not required for the study on human participants in accordance with the local legislation and institutional requirements. Written informed consent to participate in this study was provided by the participants or the participants’ legal guardian/next of kin.

## Author Contributions

AL and PL contributed to the conception of the project, carried out the annotations, and wrote the first draft of the manuscript. AL performed all the interviews. AL, EB, and PL designed and performed the quantitative study. EB performed the statistical analysis, revised the manuscript, and wrote the new sections. All the authors contributed to the initial manuscript and its revision, approving the submitted versions.

## Conflict of Interest

The authors declare that the research was conducted in the absence of any commercial or financial relationships that could be construed as a potential conflict of interest.

## Footnotes

1 https://www.finalemusic.com

2 https://provalisresearch.com/products/qualitative-data-analysis-software
